# Molecular architecture and gating mechanisms of the *Drosophila* TRPA1 channel

**DOI:** 10.1038/s41421-023-00527-1

**Published:** 2023-04-04

**Authors:** Xiaofei Wang, Yawen Li, Hong Wei, Zhisen Yang, Rui Luo, Yongxiang Gao, Wei Zhang, Xin Liu, Linfeng Sun

**Affiliations:** 1 Department of Neurology, The First Affiliated Hospital of USTC, MOE Key Laboratory for Membraneless Organelles and Cellular Dynamics, Hefei National Research Center for Interdisciplinary Sciences at the Microscale, Biomedical Sciences and Health Laboratory of Anhui Province, Division of Life Sciences and Medicine, University of Science and Technology of China, 230027 Hefei, China; 2 School of Life Sciences, IDG/McGovern Institute for Brain Research, Tsinghua University, 100084 Beijing, China; 3 Tsinghua-Peking Center for Life Sciences, 100084 Beijing, China; 4 CAS Centre for Excellence in Molecular Cell Science, University of Science and Technology of China, 230027 Hefei, China

**Keywords:** Cryoelectron microscopy, Ion channel signalling

## Abstract

The transient receptor potential channel subfamily A member 1 (TRPA1) ion channel is an evolutionary conserved polymodal sensor responding to noxious temperature or chemical stimuli. Notably, the thermosensitivity of TRPA1 varies among different species or even different isoforms in the same species. However, the underlying molecular basis of its thermo-gating remains largely unknown. Here, we determine the structures of a heat-sensitive isoform of TRPA1 in *Drosophila melanogaster* in two distinct conformations with cryo-samples prepared at 8 °C. Large conformational changes are observed in the ankyrin repeat domain (ARD) and the coiled-coil domain between the two states. Remarkably, all 17 ankyrin repeats are mapped in the newly resolved conformation, forming a propeller-like architecture. Two intersubunit interfaces are identified in the amino (N)-terminal domain, and play vital roles during both heat and chemical activation as shown by electrophysiological analysis. With cryo-samples prepared at 35 °C, only one conformation is resolved, suggesting possible state transitions during heat responses. These findings provide a basis for further understanding how the ARD regulates channel functions, and insights into the gating mechanism of TRPA1.

## Introduction

TRPA1 is a member of the transient receptor potential (TRP) superfamily with a symbolic cytosolic domain formed by a particularly large number of ankyrin repeats (ARs)^1^. It is abundantly expressed in nociceptive nerve fibres, as well as a variety of non-neuronal tissues^2–4^. TRPA1 has been identified as a primary sensor of a variety of exogenous irritants as well as endogenous molecules to signal pain and inflammation^3,5–9^. Thus, targeting TRPA1 is a promising approach for the development of analgesic and anti-inflammatory drugs. In addition to noxious compounds, TRPA1 is involved in thermosensation^2,10–14^. TRPA1 in some insects, birds and snakes has been shown to be heat-sensitive and plays pivotal roles in the avoidance of nociceptive heat or detection of infrared radiation^15–18^. In mammals, TRPA1 was initially considered to be a cold sensor^2^. However, this has become more controversial, as evidence has shown that TRPA1 can also mediate heat sensation^19–21^. Nonetheless, interspecies differences undoubtedly exist with respect to the channel’s thermosensitivity^8,22–24^. Several structural domains have been identified contributing to temperature sensing, in particular the divergent N-terminal AR-containing region^22,25,26^. Replacing part of the ARs in human TRPA1 (hTRPA1) with that from the heat-sensitive orthologue in rattlesnake or *Drosophila* confers heat sensitivity to the channel^25^. More strikingly, a single point mutation of mouse TRPA1 in the sixth AR can turn the channel from cold sensitive to heat sensitive^27^. As proposed in a thermodynamic framework, the thermosensitivity of TRP channels may arise from the difference in heat capacities for the open and closed states^28^. How the ARD as well as other regions contributes to the capacity changes and thermal sensation is merely understood.

Extensive analyses using single-particle cryo-electron microscopy (cryo-EM) have revealed the structure of hTRPA1 and how it binds to different ligands, including antagonists, and covalent or noncovalent agonists^29–33^. An open state structure of hTRPA1 upon chemical activation reveals that reactive cysteines, notably C621 and C665 of hTRPA1 located in the nexus region (also known as the linker domain), are modified by covalent agonists, leading to rearrangement of an activation-loop (A-loop) and subsequent changes in the TRP helix and gate-forming helices^31^. In the amino (N)-terminus of all up-to-date hTRPA1 structures, intrinsic flexibilities dictate that only the last five (AR12–AR16) of the predicted sixteen ARs are well defined. In addition to electrophile and thermal sensation, the ARD of TRPA1 has been suggested to be the modulation site for intracellular calcium and protein kinase A or C (PKA/PKC)-mediated phosphorylation^8^. How different modulators act on the ARD is incompletely understood, and a high-resolution structure of the full architecture of this domain is desirable to provide more mechanistic insights. Besides, since only the human TRPA1’s structure has been determined, how TRPA1 channels in other species look like remains to be investigated.

In *Drosophila*, at least four functional archetypal TRPA1 isoforms generated through alternative splicing have been identified, namely, dTRPA1-A/B/C/D (a dTRPA1-E isoform is also reported and may be a nonfunctional splicing byproduct)^34,35^. While the four isoforms exhibit similar responses to electrophilic agonists, they exhibit distinct thermosensitivities, and the dTRPA1-A isoform is shown to be the most heat-sensitive to either absolute temperature or heating rate^18,34,35^. The only differences in amino acid sequences of the four isoforms lie in the N-terminus (62 amino acids) and the allosteric nexus region (~36 amino acids). Both regions may contribute to temperature responses by defining the activation threshold, the temperature coefficient as indicated by the *Q*_*10*_ value (defined as the fold change of current per 10 °C temperature change), or the degree of sensitivity to the heating rate^18,34–36^, while the underlying mechanism remains unclear.

In this study, we set out to determine the structure of the dTRPA1-A isoform and examine its thermosensitivity and chemosensitivity with mutagenesis and electrophysiological analyses, with a focus on the thermal-gating mechanisms.

## Results

### Structure determinations for the dTRPA1-A isoform

**Fig. 1:**
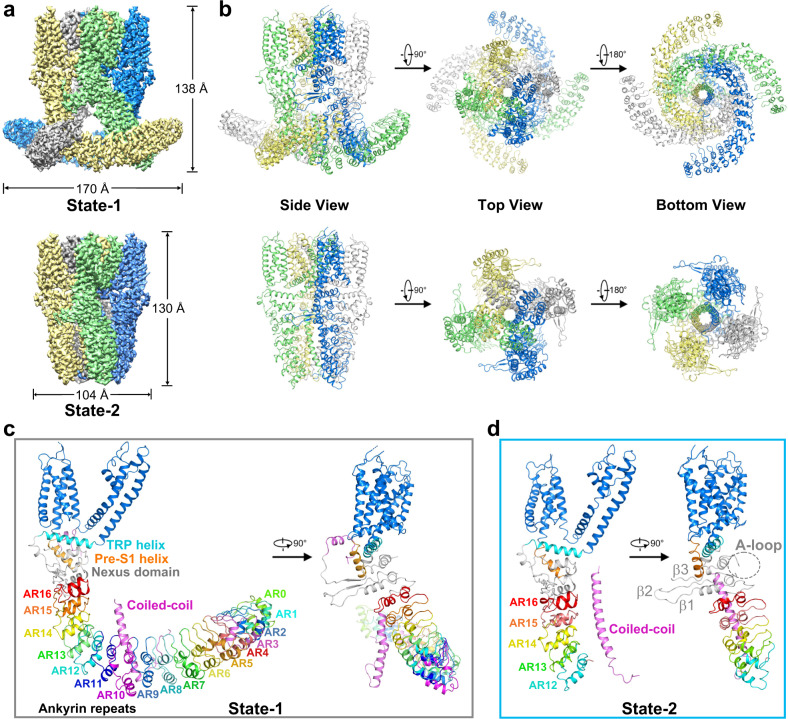
Structure determination of the *Drosophila* TRPA1-A isoform. **a** EM density maps of dTRPA1-A in two distinct states, with an overall resolution of 3.2 Å for state-1 (upper) and 3.0 Å for state-2 (lower). A side view is shown here with each of the four subunits colour-coded. **b** Cartoon representations of the dTRPA1-A structures in two states. Three views are shown here with each subunit colour-coded. **c** Overview of the subunit structure of dTRPA1-A in state-1. Seventeen ARs, AR0–AR17, are shown in this state with colour coding. The transmembrane domain is shown in marine blue, and the TRP helix and pre-S1 helix are coloured cyan and orange, respectively. The coiled-coil domain is shown in magenta. **d** Overview of the subunit structure of dTRPA1-A in state-2. The last five ARs, AR12–AR16, are visualised in this state with colour coding. The A-loop is indicated by a grey dashed circle. The three β-strands in the nexus domain are labelled β1–β3.

The full-length dTRPA1-A protein contains 1197 amino acids. To get enough protein for structural studies, the cDNA of dTRPA1-A was subcloned into a pEG BacMam vector with a designated N-terminal FLAG-tag for affinity purification^37^. The protein was expressed in suspended HEK293F cells and purified using the detergent glyco-diosgenin (GDN). The protein yield was relatively low, with less than 30 μg/L of cells. Nonetheless, the protein showed fairly good behaviour in gel filtration. Samples were prepared for cryo-EM single-particle analysis at a regular temperature of 8 °C (Supplementary Fig. S1a, b). During 3D ab initio reconstruction and classification, two good classes with distinct conformations appeared and were selected for subsequent data processing. By further classification and refinement, we obtained two EM maps with overall resolutions of 3.2 Å and 3.0 Å, respectively (Fig. 1a; Supplementary Fig. S1c–j). One of the maps, which was much elongated and outstretched in its N-terminal region, accounted for 12.0% of all selected good particles after 2D classification (named state-1 hereafter). The other one, as with those determined for hTRPA1 in which only a partial cytosolic domain was observed, accounted for 22.2% (named state-2 hereafter). Apparently, the two maps could not be merged, especially in the cytoplasmic region (Supplementary Fig. S1j, inner panel).

De novo model building was carried out for both maps (Fig. 1b). For state-1, 1053 residues (88.0% of the total number of residues) were structurally built with 953 residues assigned (Fig. 1c; Supplementary Fig. S2a). The N-terminus starts from residue number 58, with most of the ARs faithfully assigned. Unfortunately, the two aforementioned fragments leading to different temperature responses of alternative dTRPA1 isoforms, one in the N-terminus and the other in the loop of the nexus region, are missing in both structures, precluding further structural analysis. As with hTRPA1, the transmembrane domain of each subunit consists of six transmembrane α-helices (S1–S6), with S1–S4 forming the voltage-sensor-like domain (VSLD) and S5–S6 forming the pore domain organised in a domain-swapped manner. The juxtamembrane allosteric nexus region is formed by two helix-turn-helix motifs, a three-stranded antiparallel β-sheet, the pre-S1 helix and the TRP helix. The carboxyl (C)-terminus forms a four-helix coiled-coil domain located under the ion permeation pore. For state-2, 591 residues of each monomer (49% of the total number of residues) were built with 585 residues reliably assigned (Fig. 1d and Supplementary Fig. S2b). The cytosolic N-terminus starts from residue number 477 and most of the ARD is unmodelled due to vague EM densities.

We also prepared cryo-EM samples of dTRPA1-A after pre-incubating the same batch of purified protein at 35 °C for 5 min. Data collection and processing revealed the presence of only one major high-resolution 3D class. The overall resolution of the EM map was determined at 3.4 Å (Supplementary Fig. S1k, i). Structure alignments revealed that it is almost identical to the state-2 structure of dTRPA1-A determined at 8 °C, with an RMSD of 0.714 Å (Supplementary Fig. S1m). Hence, we focus on structural analysis of the two structures obtained at 8 °C.

### Architecture of dTRPA1-A isoform

The ARD of the dTRPA1-A isoform shares ~30% sequence identity with that of hTRPA1 (Supplementary Fig. S3). The N-terminus of the human TRPA1 channel is predicted to contain 16 typical ARs (excluding the first helix-turn-helix motif of the nexus domain, which is assigned as the 17th AR in some studies)^38^. In the state-2 structure of dTRPA1-A, like all hTRPA1 structures determined, only the last five ARs (AR12–AR16) were reliably assigned (Fig. 1d and Supplementary Fig. S2b). However, in state-1, the ARD is prominently stabilised in a four-leaf propeller-shaped structure, and the high local resolutions allow modelling of 17 ARs in total (AR0–AR16; Fig. 1c). The first AR is termed AR0 in compliance with the naming of hTRPA1. Most amino acid residues of the ARs are faithfully built with side chains clearly assigned, except for AR0–AR1 and the first helix of AR2 (Supplementary Fig. S2a). For hTRPA1, two speculative models have been proposed for the first 11 ARs, a wing-shaped structure and a propeller-shaped structure similar to the architecture of the ARD in our state-1 structure of dTRPA1-A isoform, and the propeller orientation was favoured based on the movement of the crescent-shaped density observed in negative-stain particles^29^. Whether hTRPA1 adopts such a stable conformation under certain conditions remains to be investigated.

Among the AR-containing TRP channels, TRPA and TRPN families have distinctively large numbers of ARs in the N-terminus. In the structure of *Drosophila* NOMPC (dNOMPC, the founding member of the TRPN family), 29 ARs form a helical spring-like architecture^39^. The overall trend for the 17 ARs in the dTRPA1 state-1 structure is distinct from that of the dNOMPC structure (Supplementary Fig. S4a). From AR0 to AR16, the ARs of dTRPA1 state-1 first goes downwards and then moves upwards, with AR10 being the turning point (Supplementary Fig. S4b). In particular, the amino acid sequence of AR10 in dTRPA1-A is the longest among all 17 ARs, containing 41 residues compared to 33 residues in a typical AR (Supplementary Fig. S3). This is reflected in the long loop after the second α-helix in AR10, from Q428 to S442. Besides, the C-terminus of the second α-helix in AR10 is dramatically tilted towards AR9 (Supplementary Fig. S4b).

**Fig. 2:**
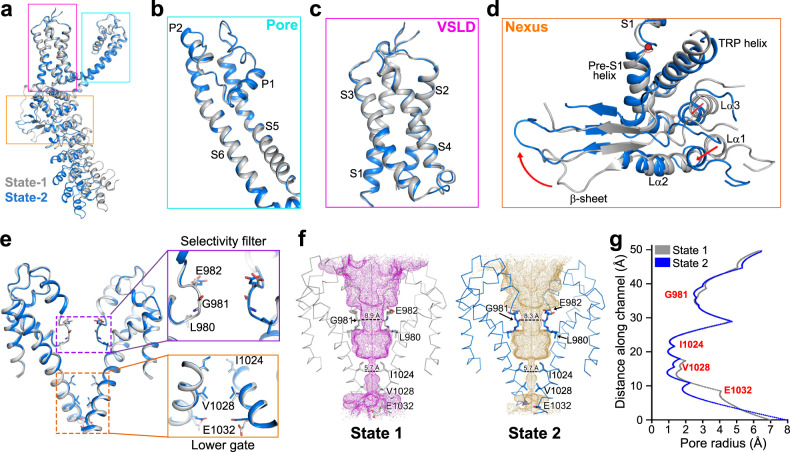
Both dTRPA1-A structures are in the nonconductive state. **a** Alignment of the monomeric dTRPA1-A structures of two states. Monomers from state-1 and state-2 are coloured grey and marine blue, respectively. **b** Structure alignments of the pore region between two states. **c** Structure alignments of the VSLD region between two states. **d** The nexus region exhibits a large rotation around the pivotal point (shown as a red circle) at the joint of preS1 and S1 helices. **e** Comparison of the pore region of dTRPA1-A in two states. Zoomed views of the upper selectivity filter and the lower gate are shown from two perpendicular angles. The constriction-site-forming residues are shown as sticks. **f** Pore depiction of dTRPA1-A in state-1 and state-2. A ribbon diagram is shown for the pore region from diagonally opposed subunits. Side chains of the constricting sites are shown as sticks. The upper gate is formed by residues L980, G981 and E982, and the lower gate is formed by I1024 and V1028. The ion conduction pore, calculated using HOLE2^57^, is shown as magenta or orange mesh for state-1 or state-2, respectively. **g** Calculated pore radius along the channel. The lower gates have almost identical radii, approximately 1 Å, indicating a nonconductive state for both structures.

Structure alignment of the monomers of the two states of dTRPA1-A isoform reveals a root-mean-square deviation (RMSD) of 3.0 Å (535 Cα atoms aligned; Fig. 2a). The pore region of the transmembrane domain aligns well except for trivial movements observed in the loop region between the upper half of S5 and pore helix 1 (P1 helix) (Fig. 2b). The VSLD is also nearly identical between two states (Fig. 2c). Substantial movements were observed for the nexus region and the ARD (Fig. 2d). From state-1 to state-2, the nexus region has a large degree of rotation around the pivotal point at the joint of pre-S1 and S1 helix (Fig. 2d; Supplementary Video S1). The β-sheet region moves upwards, accompanied by the downward shifts of the two helix-turn-helix motifs and the ARD. Strikingly, when we align the nexus domain and the ARD alone of the two states, it merges perfectly well, with an RMSD of 0.7 Å (259 Cα atoms aligned), suggesting that the nexus domain and the last five ARs move in a nearly rigid-body manner during state transitions (Supplementary Fig. S5a).

The selectivity filter of the dTRPA1-A isoform is composed of L980, G981 and E982 (Fig. 2e, f). The upper gate is almost identical in either state, except for slight differences in the side chains of E982 (Fig. 2e). The narrowest sites are formed by the backbone Cα atom of G981 with distances of 8.9 Å and 8.3 Å between the diagonally opposed subunits, respectively, and hydrated radii of ~2.5 Å for both states (Fig. 2f, g). The filters are wide enough to allow the passage of Ca^2+^ ions. The lower gate of dTRPA1 is formed by the side chains of residues I1024 and V1028. I1024 merges perfectly for the two states, while V1028 exhibits tiny shifts due to fluctuations in the lower half of S6 and the succeeding TRP helix (Fig. 2e). The shortest distance between diagonally opposed subunits and the calculated pore radii are approximately 5.7 Å and 1 Å for either state, indicating completely sealed lower gates (Fig. 2f, g). Therefore, the two conformations observed for dTRPA1-A isoform are both in the pore-closed, nonconductive state.

Although sharing the same domain composition between dTRPA1-A isoform and hTRPA1, structural alignments of the two dTRPA1-A structures with hTRPA1 in the closed or activated state reveal large variations in the VSLD, nexus region and ARD (Supplementary Fig. S5b–f). A similar architecture is observed for the pore region between the two structures of dTRPA1-A and the closed state of hTRPA1 (Supplementary Fig. S5d). Furthermore, when we separately align the last five ankyrin repeats, AR12–AR16, they all adopt a nearly identical conformation, indicating that these ARs move synchronously as an independent module during structural transitions between different functional states (Supplementary Fig. S5g).

### An interfacial helix plays a pivotal role during state transitions and channel activation

**Fig. 3:**
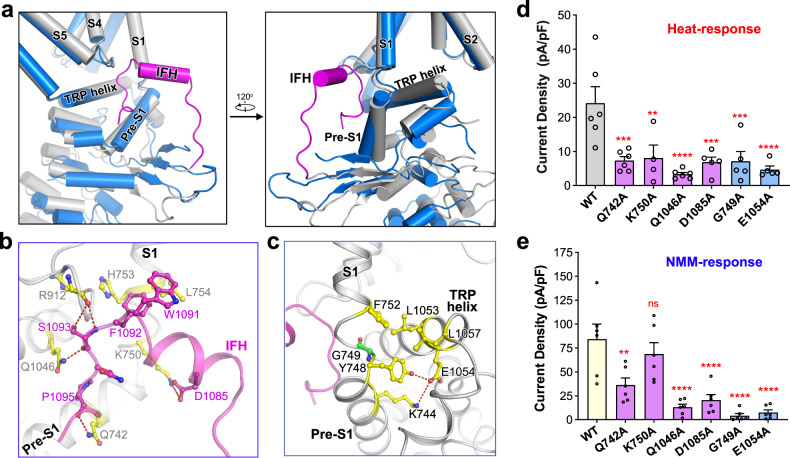
IFH is stabilised in the state-1 structure of the dTRPA1-A isoform. **a** Zoomed views of the structure alignment between monomeric dTRPA1-A in two states. Monomers from state-1 and state-2 are coloured grey and marine blue, respectively. The IFH observed in state-1 is shown in magenta. **b** Zoomed views of the IFH and the IFH-loop. **c** Interactions between the pre-S1, S1 and TRP helices. Side chains of the surrounding structure are coloured by atom type, with carbon shown in yellow. Hydrogen bonds are indicated by red dashed lines. **d** Heat-induced current densities for WT dTRPA1-A and the IFH mutants. Independent experiments were repeated for at least four times for each construct (*n* = 6 for all, except K750A where *n* = 4; and D1085A, G749A and E1054A where *n* = 5). **P = 0.002, ***P < 0.0005, and ****P < 0.0001 for WT versus mutants (one-way ANOVA with Dunnett’s multiple comparisons test). Data are mean ± s.e.m. **e** NMM-induced current densities at −60 mV for WT dTRPA1-A and the IFH mutants. Independent experiments were repeated six times for each construct (*n* = 6 for all). ns not significant, **P = 0.0016, and ****P < 0.0001 for WT versus mutants (one-way ANOVA with Dunnett’s multiple comparisons test). Data are mean ± s.e.m.

In the state-1 structure of the dTRPA1-A isoform, part of the linker between the β-sheet region and the C-terminal coiled-coil domain was clearly determined (Supplementary Fig. S2a). A short helix is assigned in the vicinity of the S1 and S4–S5 linkers, termed the interfacial helix (IFH) as in the hTRPA1 structures (Fig. 3a)^30^. However, in the state-2 structure, the EM density of this region was too nebulous to allow any structural assignment. In state-1, IFH and the succeeding loop (named IFH-loop) insert into a pocket formed by the pre-S1 helix, S1, TRP helix and S4–S5 linker and are stabilised by hydrogen bonding and ionic and hydrophobic interactions with adjacent elements (Fig. 3b and Supplementary Fig. S5h). Specifically, D1085 in the IFH forms a salt bridge with K750 in the pre-S1 helix. The hydrophobic, aromatic rings of W1091 and F1092 on the C-terminus of IFH closely pack against L754 in S1 and F913 in the S4–S5 linker. S1093 in the IFH-loop forms hydrogen bonds with the main-chain carbonyl group of R912 in the S4–S5 linker and the side chain of Q1046 in the TRP helix. The backbone carbonyl group of P1095 forms a hydrogen bond with Q742 in the pre-S1 helix. Such an extensive interaction network is absent in hTRPA1 structures due to the relatively loose packing^30^. Compared with the state-2 structure of dTRPA1-A, these interactions and the insertion of the IFH-loop cause pre-S1 to rotate around the pivot at the joint of pre-S1 and S1 by 12˚ (Fig. 3a, c; Supplementary Fig. S5i). Notably, a glycine residue lies in the pivot region, G749, providing much freedom of conformational changes (Fig. 3c). Rotation of pre-S1 is coupled to, or may be a direct trigger of the aforementioned rigid-body movements observed for the nexus region and ARD and results in large conformational changes between state-1 and state-2 structures of the dTRPA1-A isoform. In addition, robust interactions are found between the S1, pre-S1 and TRP helices, resulting in the orchestrated movements, as observed in the rotation of TRP helix by 10˚ (Fig. 3c and Supplementary Fig. S5i). Specifically, E1054 in the TRP helix forms a hydrogen bond with Y748 and an ionic interaction with K744 in the pre-S1 helix. Hydrophobic interactions are also found between L1053/L1057 in the TRP helix and F752 in the S1 helix.

In the structure of hTRPA1 bound to the covalent agonist JT010, the IFH forms an interfacial cavity with surrounding elements that accommodates a lipid molecule, while in the benzyl isothiocyanate (BITC)-bound hTRPA1 structure, the lipid molecule is missing due to the structural shift of IFH and reduced cavity size^30^. Compared with the hTRPA1 structures, the IFH in the state-1 structure of dTRPA1-A packs much more tightly to nearby helices. As shown by structure alignment, the IFH in the state-1 dTRPA1-A structure is positioned even closer to the S1 helix, pre-S1 helix and S4–S5 linker (Supplementary Fig. S5j). Consequently, no lipid molecules were observed in the interfacial cavity.

To inspect the role of the structural findings during the thermal activation of dTRPA1-A isoform, we set up an electrophysiology analysis system, generated mutants of the key residues and examined their channel activities when exposed to heat stimuli. Expression vectors for the wild-type (WT) dTRPA1-A or the mutants were transfected into *Drosophila* S2 cells. As shown by whole-cell patch-clamp recording, cells expressing WT dTRPA1-A exhibited dramatic current responses as the temperature increased (Supplementary Fig. S6a). The current density was approximately 24 pA/pF, and the Arrhenius plot showed a *Q*_*10*_ value of 18.81 with a transition temperature of 31.2 °C (Supplementary Fig. S6b and Table S2). For residues mediating the interactions between the IFH region and the surrounding elements, Q742 in the pre-S1 helix, K750 in the linker of pre-S1 and S1 helices, and Q1046 in the TRP helix, were mutated to alanine, respectively. Cells expressing the Q742A, K750A or Q1046A mutant all had largely impaired responses to heat stimuli (Fig. 3d; Supplementary Fig. S6d, e). Q1046A conducted little current (less than 15% of the WT) and had a much lower *Q*_*10*_ value (2.22, see Supplementary Table S2), suggesting that this mutation almost completely abolished the temperature sensitivity of the channel. Q742A and K750A retained only partial heat-evoked responses with highly reduced current densities (~33% of the WT) and *Q*_*10*_ values (Fig. 3d; Supplementary Fig. S6d, e and Table S2). Expression of the mutants in cells were comparable to the WT channel as examined by western blot, suggesting that the impaired responses of the mutants were not due to changes in the protein level (Supplementary Fig. S7). These results confirm that the IFH helix and IFH-loop are essential for the thermal activation of dTRPA1-A. The pivot-forming residue of pre-S1 rotating, G749 of dTRPA1, is conserved in hTRPA1. Notably, the G749A mutant had much impaired currents upon heat treatment (~30% of the WT; Fig. 3d and Supplementary Fig. S6d). The interactions between pre-S1 and TRP helices are also needed for channel activation, as the E1054A mutant retained little heat response (~20%) compared to the WT (Fig. 3d; Supplementary Fig. S6d).

We asked whether the IFH helix and IFH-loop are also involved in the chemical activation of dTRPA1. N-methyl maleimide (NMM), an electrophile that can covalently modify cysteine residues and specifically activate TRPA1 in the membrane, was used to treat *Drosophila* S2 cells^18,40^. As shown by whole-cell patch-clamp recordings, cells transfected with the WT TRPA1-A isoform responded robustly to NMM treatment (Supplementary Fig. S6c). We then tested the NMM response of the mutants generated. For the residues mediating the interactions with IFH and the IFH-loop, while K750A had slightly reduced NMM induced current density, the Q742A or Q1046A mutants had much impaired channel activities (Fig. 3e). The G749A and E1054A mutant, located in the preS1–S1 linker and TRP helix, respectively, also had little channel activities under NMM stimuli (Fig. 3e).

Together, these results reinforce the critical role of IFH and IFH-loop, the rotation of pre-S1 and orchestrated movements of TRP helix and ARD during the heat and chemical responses of dTRPA1-A isoform.

### The C-terminal coiled-coil domain undergoes an up-and-down movement during state transitions

**Fig. 4:**
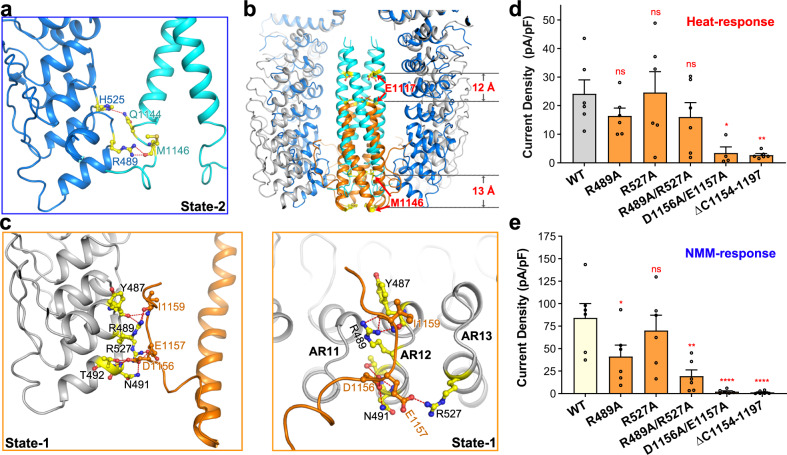
The C-terminal coiled-coil domain of dTRPA1-A isoform undergoes a large movement during state transitions. **a** Interactions between the C-terminal coiled-coil domain and surrounding ARD in state-2 structure of dTRPA1-A isoform. The C-terminal coiled-coil domain and ARD of state-2 are coloured cyan and marine, respectively. **b** Compared with the state-2 structure, the coiled-coil domain has a large downward movement with ~12–13 Å in the state-1 structure. The C-terminal coiled-coil domain and ARD of state-1 are coloured orange and grey, respectively. **c** Interactions between the C-terminal coiled-coil domain and surrounding ARD in state-1 structure of dTRPA1-A isoform. Two views are shown here. **d** Heat-induced current densities for WT dTRPA1-A and the C-terminal mutants. Independent experiments were repeated for at least four times for each construct (*n* = 6 for all, except D1156A/E1157A where *n* = 4). ns not significant, *P = 0.0267, and **P = 0.0088 for WT versus mutants (one-way ANOVA with Dunnett’s multiple comparisons test). Data are mean ± s.e.m. **e** NMM-induced current densities at −60 mV for WT dTRPA1-A and the C-terminal mutants. Independent experiments were repeated six times for each construct (*n* = 6 for all). ns not significant, *P = 0.042, **P = 0.0013 and ****P < 0.0001 for WT versus mutants (one-way ANOVA with Dunnett’s multiple comparisons test). Data are mean ± s.e.m. For **d** and **e**, the same values for the WT channel are used as in Fig. 3d, e for consistent comparison since all the electrophysiological data were acquired using the same batch of cells.

In both structures of dTRPA1-A, the C-terminal coiled-coil domain forms the main cytosolic interactions to stabilise the complex assembly, resembling that of hTRPA1. In the state-2 structure of dTRPA1-A, the EM density of the coiled-coil domain is clear for residue assignment (Supplementary Fig. S2b). The loop following the coiled-coil helix (named C-loop) was also partially determined. Notably, this loop interacts with the surrounding ankyrin repeats domain. Hydrogen bonding is found between the side chains of Q1144 and H525 (in AR13), and between the main chain of M1146 and the side chain of R489 (in AR12) (Fig. 4a). In the state-1 structure of dTRPA1-A, the EM density is less clear, especially for the upper part of the coiled-coil domain (Supplementary Fig. S2a). Nonetheless, we were able to assign the lower part of the coiled-coil helix and the C-loop. Strikingly, compared with that of state-2 structure, the coiled-coil domain has a large downward movement with ~12–13 Å (Fig. 4b; Supplementary Video S1). Moreover, the C-loop has a different interaction profile with the ankyrin repeats domain. Briefly, D1156 interacts with N491 and T492 in AR12. E1157 forms a salt bridge with R527 in AR13. The backbone of I1159 interacts with the side chain of R489 and main chain of Y487 in AR12 (Fig. 4c).

To examine the roles of the C-terminal loop and its interacting ARs, we generated several mutants and checked their heat and NMM responses, respectively. For the heat stimuli, current density for the R489A or R527A single mutant, both located in the ARD, was comparable to or slightly reduced by ~25% compared to the WT channel, as well as for the R489A/R527A double mutant (Fig. 4d; Supplementary Fig. S6d). However, for residues in the C-loop, the D1156A/E1157A double mutant also had a dramatically impaired heat response (Fig. 4d; Supplementary Fig. S6d and Table S2). Besides, we generated a truncation mutant by deleting the C-loop (ΔC1154–1197). Like D1156A/E1157A, the heat response of this truncated protein was almost totally lost (Fig. 4d; Supplementary Fig. S6d and Table S2). For NMM treatment, the R489A/R527A, D1156A/E1157A and the Δ1154 – 1197 truncation mutants all had little response, similar to the heat treatment (Fig. 4e). Together, it suggests that the C-loop is essential for the heat activation of dTRPA1-A isoform, and interactions between the C-loop and ARD rearranges along with the conformational changes of the ARD and the up-and-down movement of the coiled-coil domain.

### Two interfaces are observed in the ARD domain of the dTRPA1-A state-1 structure between adjacent subunits

**Fig. 5:**
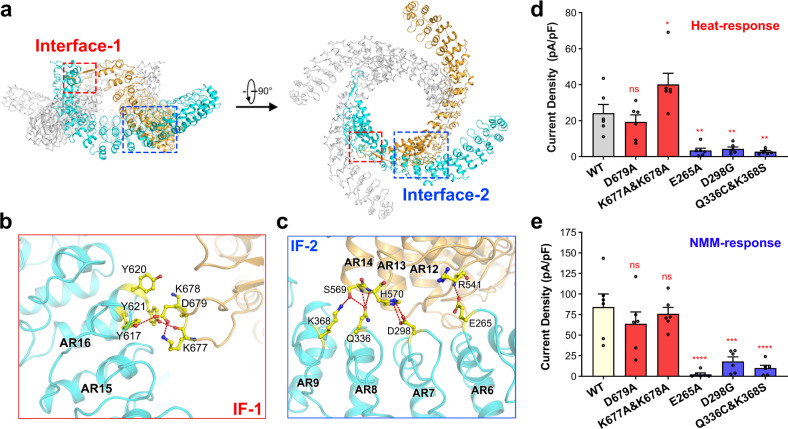
ARD adopts a unique architecture in the state-1 structure of the dTRPA1-A isoform. **a** Two interfaces, interface-1 and interface-2, are observed between adjacent subunits in the cytosolic domain of the dTRPA1-A state-1 structure, as indicated by red and blue boxes, respectively. **b** Zoomed view of interface-1 (IF-1) of the dTRPA1-A state-1 structure. Two neighbouring subunits are shown in cyan and light orange. Residues at the interface are shown in sticks and the polar contacts are indicated by red dashed lines. **c** Zoomed view of interface-2 (IF-2) of the dTRPA1-A state-1 structure. **d** Heat-induced current densities for WT dTRPA1-A and the IF mutants. Independent experiments were repeated six times for each construct (*n* = 6 for all). ns not significant, *P = 0.018, and **P < 0.003 for WT versus mutants (one-way ANOVA with Dunnett’s multiple comparisons test). Data are mean ± s.e.m. **e** NMM-induced current densities at −60 mV for WT dTRPA1-A and the IF mutants. Independent experiments were repeated six times for each construct (*n* = 6 for all). ns not significant, ***P = 0.0002, and ****P < 0.0001 for WT versus mutants (one-way ANOVA with Dunnett’s multiple comparisons test). Data are mean ± s.e.m. For **d** and **e**, the same values for the WT channel are used as in Fig. 3d, e for consistent comparison since all the electrophysiological data were acquired using the same batch of cells.

Besides the coiled-coil domain, two new interfaces are identified between neighbouring subunits in the ARD domain of the state-1 structure (Fig. 5a). One is between AR16 of one subunit and the nexus domain from the adjacent subunit (the loop between the first two β-strands), named interface-1, and the other is within the ARDs between two adjacent subunits (AR6–AR9 of one subunit and AR12–AR14 of the neighbouring subunit), named interface-2. In interface-1, the inner helix of AR16 and the loop region contribute to all of the polar interactions (Fig. 5b). In particular, the loop contains three consecutive charged residues, K677, K678 and D679. They are adjacent to three tyrosine residues in the inner helix of AR16, Y617, Y620 and Y621. Specifically, the main-chain carbonyl group of K677 in the loop region interact with the side chain of Y621 in AR16. D679 interacts with both Y617 in AR16 by forming hydrogen bonds between the side chains. K677 also interacts with D679 to stabilise the configuration of D679 (Fig. 5b). Due to this interface, this loop region in the nexus domain is stabilised to reveal clear densities for residue assignment in the EM map of state-1, in contrast to the vague densities observed in the state-2 map (Supplementary Fig. S2a, b).

Interactions within interface-2 involve more structural units (Fig. 5c). AR6–AR9 of one subunit interacts with AR12–AR14 of the adjacent subunit, mainly through polar interactions. Notably, the hairpin loop contributes to most of the interactions. In detail, E265 in the hairpin loop of AR6 interacts with the main-chain amino group of R541 in AR12. D298 of AR7 interacts with H570 of AR14, and S569 of AR14 interacts with both Q336 of AR8 and K368 of AR9 (Fig. 5c). Taking account of both interfaces, three discrete modules from each subunit contribute to the interface formation within the N-terminal cytosolic domain: AR6–AR9, AR12–AR16 and the loop between the β-sheets of the nexus domain.

We tested the roles of each interface by generating mutations to disrupt the interactions and monitored their heat and NMM responses. For interface-1, the D679A mutant had a similar current density to the WT upon heat treatment, while the K677A/K678A double mutant had a much higher current density, about two-fold of the WT channel (Fig. 5d; Supplementary Fig. S6d). An Arrhenius plot of the K677A/K678A double mutant showed a larger *Q*_*10*_ value of 23.82 and a lower transition temperature of 28.1 °C (Supplementary Fig. S6e and Table S2), both suggesting that the double mutant had an increased heat sensitivity. For interface-2, we generated three mutants, E265A, D298G, and a double point mutation, Q336C/K368S (substituting D298, Q336 and K368 with the corresponding residue in hTRPA1; Supplementary Fig. S3). As the results showed, the heat-induced currents for all mutants were significantly diminished, with an average current density for each mutant < 20% of the WT channel (Fig. 5d; Supplementary Fig. S6d and Table S2). Interestingly, during NMM treatment, for residues located in the interface-1 of the cytosolic domain, while the K677A/K678A mutant had a much-increased heat response, its NMM response was similar to the WT channel (Fig. 5e). D679A retained both the NMM and heat induced currents (Fig. 5d, e). For residues mediating the interface-2 formation, all the three mutants, E265A, Q298G and Q336C/K368S had dramatically reduced response to NMM stimuli (Fig. 5e). The above results show that while disrupting interface-1 can retain the heat or NMM responses, disrupting interface-2 almost totally abolishes the channel activity, highlighting the critical role of ARD during channel activation.

### Role of cysteine residues during channel activation of dTRPA1-A isoform

In dTRPA1-A, two cysteine residues are observed in the proposed electrophile binding pocket as with hTRPA1, formed by the first helix-turn-helix motif and the A-loop (Supplementary Fig. S8a). C650 (corresponding to C621 of hTRPA1, Supplementary Fig. S8b) is located at the first helix-turn-helix motif, and C694 (corresponding to C665 of hTRPA1) is in the A-loop. Compared with that in the hTRPA1 structures, the two cysteine residues are located much closer in either state of dTRPA1-A. Separately aligning the nexus domain of the hTRPA1 and dTRPA1-A structures revealed that the first helix-turn-helix motif in dTRPA1-A was shifted towards the upper A-loop (Supplementary Fig. S8c). To determine whether these two cysteine residues are prerequisites for chemical activation or thermal sensing of dTRPA1-A channel, we mutated C650 to serine and C694 to serine or lysine (in the dTRPA1-C isoform, C694 is substituted with a lysine residue, Supplementary Fig. S8b), and examined their channel properties. Surprisingly, for all cysteine mutants tested here, the NMM responses were retained and the current densities were comparable to the WT (Supplementary Fig. S8d). Specially, C694K had a much larger NMM-induced current density than the WT channel. Similarly, under heat treatment, all the cysteine substitutions retained most of the heat responses and had comparable current densities to the WT, suggesting that the heat response was barely affected (Supplementary Fig. S8e and Table S2). Taken together, it suggests that the dTRPA1-A isoform may have other NMM modification sites and adopt a different chemical activation mechanism compared to the hTRPA1’s activation by iodoacetamide (IA) or allyl isothiocyanate (AITC), which awaits further investigations.

## Discussion

Comparative analyses of different *Drosophila* splicing isoforms or with those from other species have mapped several regions critical to thermosensitivity, including the N-terminus, the allosteric nexus region, specific ankyrin repeats domain and the pore region^18,25,27,34,36,41^. The state-1 structure of dTRPA1-A presented here offers an opportunity to visualise the overall architecture of the ARD and a reference with which to map reported mutagenesis studies on this domain. Previous chimeric channel characterisations revealed that substitution of AR10–AR15 of hTRPA1 with that of the dTRPA1 changed hTRPA1 into a heat-sensitive channel^25^. In our state-1 structure of dTRPA1-A isoform, such an ARD module participates in the interface-2 formation and as the electrophysiology results show, this interface is critical to the heat sensitivity of dTRPA1. Another work using a vast random mutant screening method suggests that a single ankyrin repeat, AR6, is a key modulator of thermal activation in mouse TRPA1^27^. Three residues located in AR6 are identified to be essential, S250, M258 and D261 in mouse TRPA1 (corresponding to G276, L284 and K287 in dTRPA1-A isoform). Three single point mutations of mouse TRPA1, S250N, M258L and D261G, separately, can turn the channel into heat activated^23^. However, a reciprocal mutant in dTRPA1, G276N, almost totally abolishes it^27^. In the state-1 structure, G276 locates at the turning loop between the inner helix and outer helix of AR6, lying at the interface-2. L284 and K287 locates at the C-terminus of the outer helix (Supplementary Fig. S9a). Mutating G276 to asparagine, which has a larger side chain, may lead to structural disruptions of the loop region and the formation of interface-2, thus affecting the overall architecture of ARD and heat signal transition to the transmembrane gates.

Another interesting observation is that the C-terminal coiled-coil domain undergoes a large conformational change between state-1 and state-2. This domain has been implicated in mediating subunit interactions and polyphosphate association (such as inositol hexakisphosphate (InsP_6_)) in hTRPA1 and is clearly resolved in all hTRPA1 structures^29^. As our results show, such conformational changes and the accompanied C-loop rearrangements also affect channel activation during either heat or chemical stimuli in dTRPA1-A isoform. Besides, we also tested if the C-terminal loop truncation would affect the channel activation of hTRPA1. The results showed that compared with WT hTRPA1, the C-terminal loop truncation (ΔC1080–1119) had a completely abolished response to AITC treatment, the same as the result observed in the dTRPA1-A mutant (Supplementary Fig. S6f), suggesting conserved roles of the C-terminus in channel activation in TRPA1 homologues. In addition to TRPA1, the coiled-coil domain is also found in other TRP channels, such as the transient receptor potential melastatin (TRPM), transient receptor potential channel (TRPC) and transient receptor potential polycystic (TRPP) families (Supplementary Fig. S9b)^42^. In the cold-activated melastatin receptor channel TRPM8, the coiled-coil domain was suggested to undergo a cold-induced folding process coupled to channel opening, serving as a key structural component of the temperature sensor^43^. In a broader spectrum, the four-helix bundle structure is also present in some voltage-gated potassium channels (such as KCNQ channels) and bacterial voltage-gated sodium channels (BacNa_V_s) (Supplementary Fig. S9b)^44,45^. Structural and functional studies of BacNa_V_s from different species reveal that the upper part (defined as the “neck”) of the four-helix bundle exhibits the most structural diversity and undergoes heat-dependent unfolding upon channel activation, in contrast to that of TRPM8^45^. Together, these results suggest that the C-terminal four-helix bundle is not only critical to channel assembly but also essential to channel gating. Besides local folding or unfolding process, the global large movement observed here for this four-helix bundle in dTRPA1 also plays an indispensable role during channel activation.

The cryo-EM technique leads to the identification of two distinct states of the dTRPA1-A isoform in the same sample prepared at 8 °C, while only state-2 structure remaining at 35 °C. The electrophysiology analysis reveals that dTRPA1-A isoform can be activated above ~30 °C (Supplementary Fig. S6a, b). Missing of the state-1 structure at the high temperature suggests that it might be a resting or pre-sensitised state prior to heat stimuli. Meanwhile, structural studies of the thermal sensitive TRPV3 channel, which also has a cytosolic ankyrin repeats domain, reveal that the channel may undergo two steps upon heat stimuli: sensitisation and opening^46^. During the sensitisation step, large conformational changes are observed in the cytosolic ankyrin repeats domain, reminiscent of the state-1 and state-2 transitions of dTRPA1-A isoform presented here. While during the channel opening step, small local rearrangements occur in the gating helices. Based on our structural analysis of dTRPA1-A isoform under low and high temperatures, and reported structures of thermal sensitive TRPV family members, we propose a possible model describing the two states transitions of the dTRPA1-A isoform (Supplementary Fig. S9c). The state-1 structure might represent an initial closed, or resting state of dTRPA1-A isoform. It equilibrates with or turns into a thermally sensitised, preopen state (state-2) as the temperature rises. The IFH dissociates from the transmembrane region, and the ARD and nexus domain rotates around the joint of pre-S1 and S1 helices. Interactions between the ARD and the C-terminal loop reorganise and the coiled-coil domain moves upwards, much closer to the pore. The channel opens when the temperature further increases above the transition threshold, probably through local structural movements similar to those observed in hTRPA1 during chemical activation (Supplementary Fig. S9c). However, we still cannot exclude other plausible gating models and further studies are needed to fully unveil the gating mechanism of TRPA family channels, particularly by obtaining a heat-activated open state structure of the dTRPA1-A isoform or other thermally sensitive orthologues. Due to vague EM densities, the two elements leading to different temperature responses of alternative dTRPA1 isoforms, the starting N-terminus region and the loop of the nexus region, are missing in both structures. How they are involved in the thermal sensing awaits investigated. Additionally, whether an architecture similar to the state-1 structure of the dTRPA1-A isoform exists in hTRPA1 and other homologues needs to be elucidated. Nontheless, our determined structures of dTRPA1-A isoform provide more molecular insights into the TRPA channels, and set up a framework for further analysis of cross-species variations in thermal sensation and gating mechanism.

## Methods

### Protein expression and purification

The original coding sequence for the *Drosophila* full-length dTRPA1-A isoform was a gift from the William Daniel Tracey lab in Indiana University and was subcloned into pEG BacMam vector with an N-terminal FLAG–tag^37^. The baculovirus was generated using Sf9 cells (Invitrogen) and then applied to HEK293F cells (Sino Biological Inc.) in suspension for protein expression, cultured in SMM 293-TI medium (Sino Biological Inc.) in a cell shaker (Shanghai Zhichu Instruments) operating at 130 rpm, 37 °C and supplemented with 5% CO_2_, when the density reached 2 × 10^6^ cells/mL. After a 10 h shaking incubation at 37 °C, 10 mM sodium butyrate (Sigma Aldrich) was added into the culture and the temperature was lowered to 30 °C to boost protein expression. Transfected cells were harvested after 48 h by centrifugation at 3000 rpm. Cell pellets were resuspended in lysis buffer containing 25 mM HEPES (pH 7.4) and 150 mM NaCl. The suspension was then supplemented with 1.5% (w/v) n-Dodecyl-β-d-Maltopyranoside (DDM, Anatrace), and the protease inhibitor cocktail containing 1 mM phenylmethyl sulfonyl fluoride (PMSF, Sigma Aldrich), aprotinin (1.3 mg/mL, Sigma Aldrich), pepstatin A (0.7 mg/mL, Sigma Aldrich) and leupeptin (5 mg/mL, Sigma Aldrich). After incubation at 4 °C for 2 h, the insoluble fraction was precipitated by centrifugation at 13,000 rpm for 60 min. The supernatant was incubated with anti-FLAG M2 affinity gel (Sigma Aldrich) at 4 °C for 30 min. The resin was rinsed two times by wash buffer 1 containing 25 mM HEPES (pH 7.4), 150 mM NaCl, 2 mM Mg^2+^, 2 mM ATP disodium salt (Sangon Biotech, Shanghai) and 0.02% (w/v) glyco-diosgenin (GDN, Anatrace). Afterwards, the resin was rinsed one time by wash buffer 2 containing 25 mM HEPES (pH 7.4), 150 mM NaCl, and 0.02% GDN. The protein was eluted with wash buffer 2 plus 200 μg/mL FLAG peptide (Sigma Aldrich). After an on-ice incubation for 2 h, the eluent was concentrated using a 100-kDa cut-off Centricon (Millipore) and then applied to the Superose-6 column (GE Healthcare) in buffer containing 25 mM HEPES (pH 7.4), 150 mM NaCl and 0.02% GDN. Peak fractions were pooled together and further concentrated to approximately 9 mg ml^−1^ before cryo-EM sample preparation.

### Sample preparation and cryo-EM data acquisition

4 μL of the purified dTRPA1-A protein in GDN was placed on the glow-discharged holey carbon grid (Quantifoil Au R1.2/1.3, 300 mesh) and then applied to the Vitrobot Mark IV (FEI) operating at 8 °C or 35 °C and 100% humidity with a blotting time of 3.5 s. Grids were transferred to the Titan Krios (FEI) electron microscope operating at 300 kV equipped with a K2 Summit electron-counting direct detection camera (Gatan). Images were recorded in the super-resolution mode under a nominal magnification of 29,000× using SerialEM^47^. Defocus values varied from −1.5 to −2.3 μm. For the low temperature sample, 7574 raw micrograph stacks were collected, with a total exposure time of 5.12 s and dose-fractionated to 32 frames with a total dose of 50 *e*^*-*^ Å^−2^. For the high temperature sample, 1983 raw micrograph stacks were collected with the same setting parameters. The stacks were motion corrected with MotionCor2^48^ and then binned two-fold to a pixel size of 1.01 Å. Dose weighting was performed at the same time. Defocus values were estimated by Gctf^49^.

### Image processing

A detailed flowchart for the data processing of the low temperature sample is presented in Supplementary Fig. S1j. Briefly, 2,672,587 particles were automatically picked using RELION 3.0^50^. 1,396,814 particles were selected after 2D classification, and then subjected to a global angular search 3D classification with five classes and 50 iterations. Particles from two classes with good features, representing two distinct states, were selected and subjected to further 3D auto-refinement, 3D classification, CTF refinement and post-processing. 3D reconstruction maps with an overall resolution of 3.2 Å and 3.0 Å after postprocessing were achieved for each state, respectively. The overall resolution was estimated with the gold-standard FSC at a 0.143 criterion with a high-resolution noise substitution method^51,52^. Local resolution variations were estimated using ResMap^53^.

### Model building and refinement

The 3.2 Å and 3.0 Å reconstruction maps for two different states after postprocessing were used for de novo model building in COOT^54^. Structure refinements were carried out by PHENIX in real space^55^. Overfitting of the model was monitored by refining the model in one of the two independent maps from the gold-standard refinement approach and testing the refined model against the other map. Statistics of the 3D reconstruction and model refinement can be found in Supplementary Table S1.

### Electrophysiology

The wild-type and dTRPA1-A variants with indicated point mutations were subcloned into the pJFRC81 vector in frame with a GFP tag at the C-terminus. *Drosophila* S2 cell line (Invitrogen) was maintained in SIM SF insect medium (Sino Biological Inc.) with 10% penicillin-streptomycin in a 25 °C incubator. S2 cells were transiently transfected with pAct5C-GAL4 and the wild-type or mutant dTRPA1-A pJFRC81 plasmids using Effectene Transfection Reagent (Qiagen). Whole-cell recording of S2 cells was performed on cells with fluorescence 48 h after transfection as previously described with slight modifications^35^. Briefly, S2 cells were plated on a PDL coated coverslip, and the external solution for recording contained 120 mM NaCl, 3 mM KCl, 4 mM MgCl_2_, 1.5 mM CaCl_2_, 10 mM NaHCO_3_, 10 mM glucose, 10 mM sucrose, 10 mM trehalose, 5 mM TES and 10 mM HEPES. The intracellular solution contained 140 mM potassium gluconate, 1 mM KCl, 1 mM EGTA and 10 mM HEPES. All solutions were adjusted to pH 7.2 and 320 mOsm by sucrose. Electrodes with 6–12 MΩ resistance were used. Cells were lifted from glass coverslips after obtaining stable whole-cell recording. Current was acquired with an Axon-200B amplifier and digitised at 10 kHz by a Digidata 1550 analog-to-digital converter, filtered at 1 kHz and membrane potential was held at −60 mV. Heat stimulation was applied via the Heating and Cooling Micro-incubation Stage (ALA Scientific Instruments) with a Digital One Channel BiPolar ALA Temperature Controller (ALA Scientific Instruments) from 24 °C to 42 °C and the temperature was recorded by a probe adjacent to the recorded cells. For NMM stimulation, NMM solution was added into chamber to a final concentration of 200 μM under 24 °C.

For hTRPA1 activation by AITC, genes for the WT and mutant were subcloned into pCDNA3.1 vector. HEK293T (Sino Biological Inc.) cells were co-transfected with hTRPA1 plasmid (WT or mutant) and pEGFP-N1 plasmid using jetOPTIMUS in vitro DNA transfection reagent. The external and intracellular solutions were same as dTRPA1. Current was acquired with an Axon-200B amplifier and digitised at 10 kHz by a Digidata 1550 analog-to-digital converter, filtered at 1 kHz and membrane potential was held at −80 mV. For AITC stimulation, AITC solution was added into chamber to a final concentration of 200 μM under 24 °C.

Recorded data were analysed using the pClamp10.7 software (Molecular Devices) and Prism 6 (GraphPad). The Arrhenius *Q*_10_ and thermal threshold was calculated as previously described^17,56^. Briefly, Arrhenius plot was obtained by plotting the logarithm of the TRPA1 currents versus the inverse absolute temperature. *Q*_10_ was calculated with the equation: $Q_{10} = \left( {\frac{{R_2}}{{R_1}}} \right)^{10/(T_2 - T_1)}$, where *R*_2_ and *R*_1_ are the current at the higher temperature *T*_2_ and the lower temperature *T*_1_, respectively. The temperature transitions (thresholds) represent the intersection between linear fits to baseline. A summary of the *Q*_10_ values and transition temperatures for the WT dTRPA1-A isoform and the mutants can be found in Supplementary Table S2. Statistical analyses were performed using one-way ANOVA with Dunnett’s multiple comparisons test.

## Supplementary information

Supplementary Information

Supplementary Video S1

## Data Availability

The 3D cryo-EM density maps of dTRPA1-A isoform in two states have been deposited in the Electron Microscopy Data Bank under the accession number EMD-33895 and EMD-33896, respectively. Coordinates for the two dTRPA1-A structure models have been deposited in the PDB under the accession code 7YKR and 7YKS, respectively.
