# Supplementary material for: Molecular architecture and gating mechanisms of the Drosophila TRPA1 channel
Source: Cell Discov. 2023 Apr 4;9:36. doi: 10.1038/s41421-023-00527-1 (PMC10073219; doi:10.1038/s41421-023-00527-1)
Supplement: Supplementary file 1 — Supplementary Information [file 41421_2023_527_MOESM1_ESM.pdf]

Supplementary Information for

Molecular architecture and gating mechanisms of the *Drosophila* TRPA1 channel

Xiaofei Wang[1,5], Yawen Li[1,5], Hong Wei[1,5], Zhisen Yang[1,5], Rui Luo[2,4], Yongxiang Gao[1], Wei Zhang[2,4,6], Xin Liu[1,6] & Linfeng Sun[1,3,6]

[1] Department of Neurology, The First Affiliated Hospital of USTC, MOE Key Laboratory for Membraneless Organelles and Cellular Dynamics, Hefei National Laboratory for Physical Sciences at the Microscale, Biomedical Sciences and Health Laboratory of Anhui Province, Division of Life Sciences and Medicine, University of Science and Technology of China, Hefei 230027, China.

[2] School of Life Sciences, IDG/McGovern Institute for Brain Research, Tsinghua University, Beijing 100084, China.

[3] CAS Centre for Excellence in Molecular Cell Science, University of Science and Technology of China, Hefei 230027, China.

[4] Tsinghua-Peking Center for Life Sciences, Beijing 100084, China.

[5] These authors contribute equally to this work.

[6] To whom correspondence should be addressed. E-mail: wei_zhang@mail.tsinghua.edu.cn, lx023@ustc.edu.cn, or sunlf17@ustc.edu.cn.

This file includes:

Supplementary Figures S1 to S9

Supplementary Table S1 to S2

Supplementary Video S1

Supplementary Figures

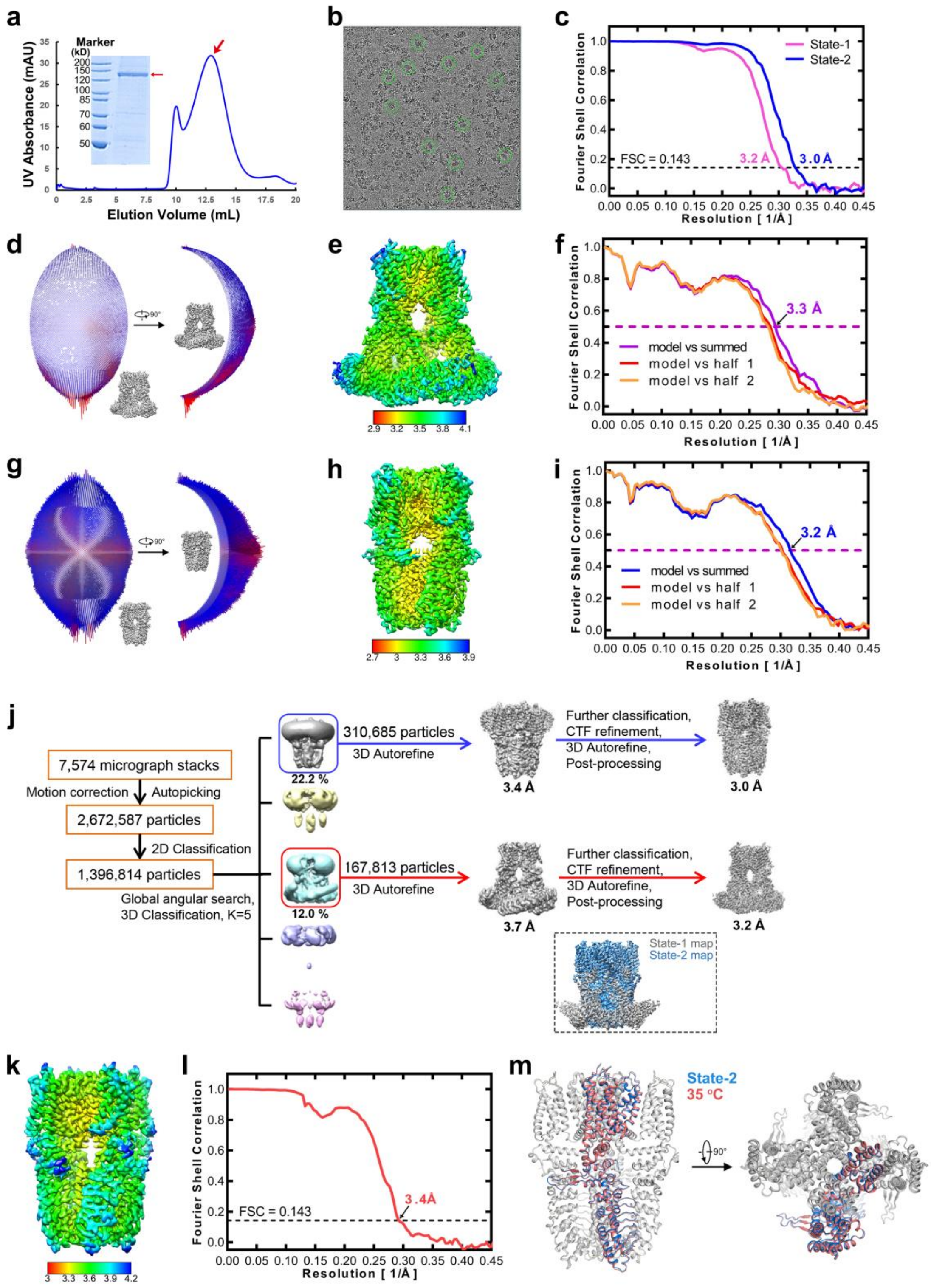

Supplementary Fig. S1 Cryo-EM analyses of the dTRPA1-A isoform.

a. A representative gel filtration analysis of the dTRPA1-A isoform. The mono-disperse peak of the dTRPA1-A isoform, indicated by the red arrow and

verified by Coomassie-blue-staining SDS-PAGE, suggests a good solution behaviour of the sample. **b.** A cryo-EM image of the dTRPA1-A isoform. Representative particles are indicated by green circles. **c.** Gold-standard Fourier shell correlation curves for the overall maps of two states. **d.** Euler angle distribution of the final 3D refinement of state-1. **e,** Local resolution map of state-1 calculated using ResMap. **f.** FSC curves of the refined model versus the overall map of state-1 that it was refined against (magenta); of the model refined in the first of the two independent maps used for the gold-standard FSC versus that same map (red); and of the model refined in the first of the two independent maps versus the second independent map (orange). **g.** Euler angle distribution of the dTRPA1-A state-2 map. **h.** Local resolution map of the dTRPA1-A state-2 map. **i.** FSC model curves of the dTRPA1-A state-2 map. **j.** Flowchart for cryo-EM data processing. Details can be found in the ‘‘Image processing’’ session in Methods. Inner panel, merge of the two EM-maps. **k.** Local resolution map of dTRPA1-A isoform captured at 35 °C, as calculated using ResMap. **l.** The gold-standard Fourier shell correlation curves for the overall map of dTRPA1-A isoform captured at 35 °C. **m.** Structure alignments between the state-2 structure captured at 8 °C (coloured marine) and the structure captured at 35 °C (coloured red).

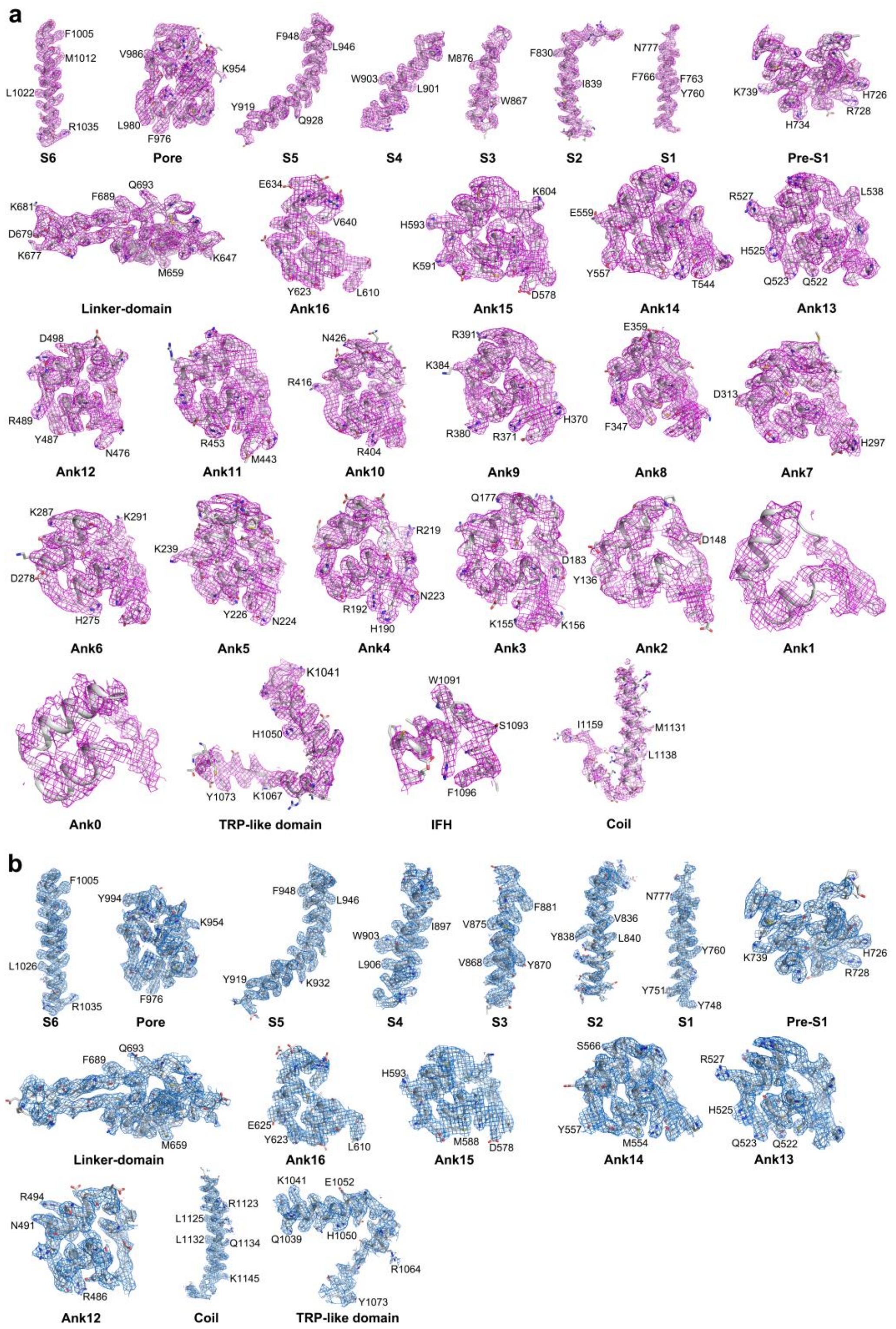

Supplementary Fig. S2 Representative EM densities for the dTRPA1-A isoform.

a. EM densities for the transmembrane helices, linker domain, seventeen ankyrin repeats, TRP helix, IFH and coiled-coil domain of state-1 are shown in magenta mesh.

b. EM densities for the transmembrane helices, linker domain, five ankyrin repeats, TRP helix and the coiled-coil domain of state-2 are shown in marine blue mesh.

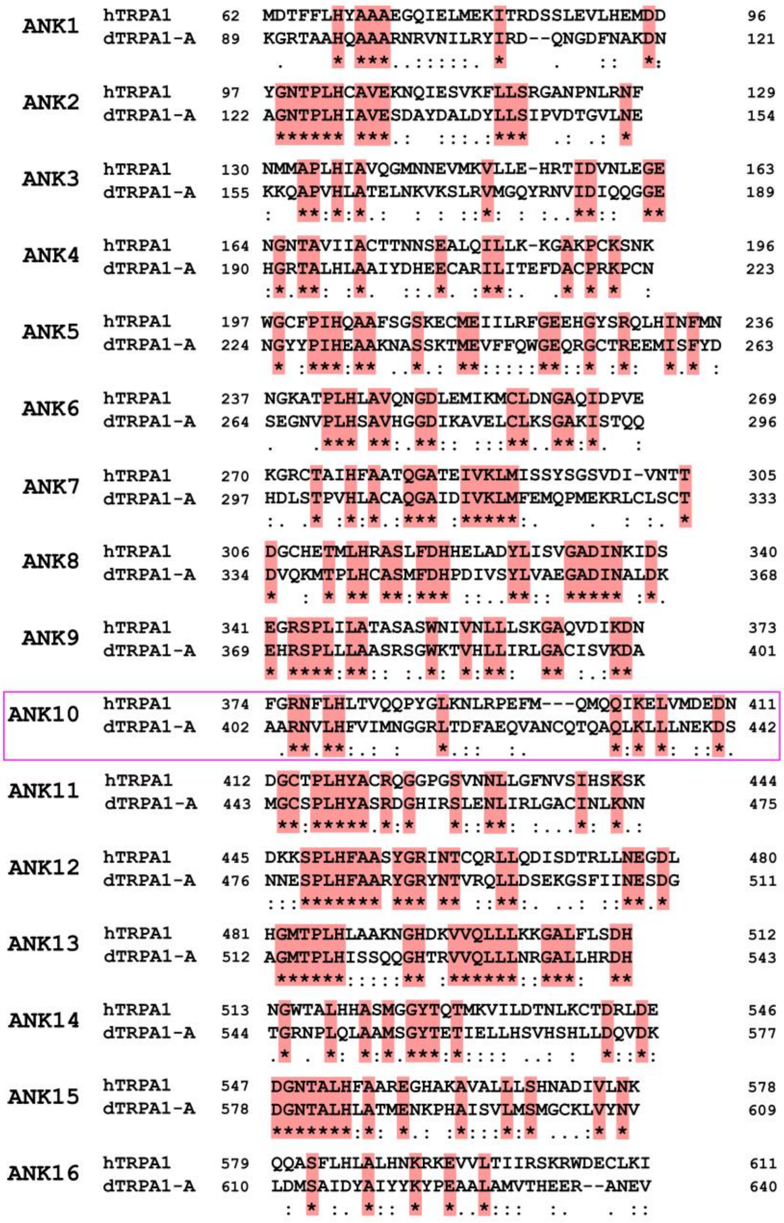

Supplementary Fig. S3 Sequence alignments of the ARD in hTRPA1 and dTRPA1-A isoform.

Shown here are pair-wise sequence alignments of the sixteen conserved ARs predicted in hTRPA1 and dTRPA1-A. The degree of conservation is indicated in a ClustalW pattern as shown on the bottom of the sequence. Identical residues are shaded red.

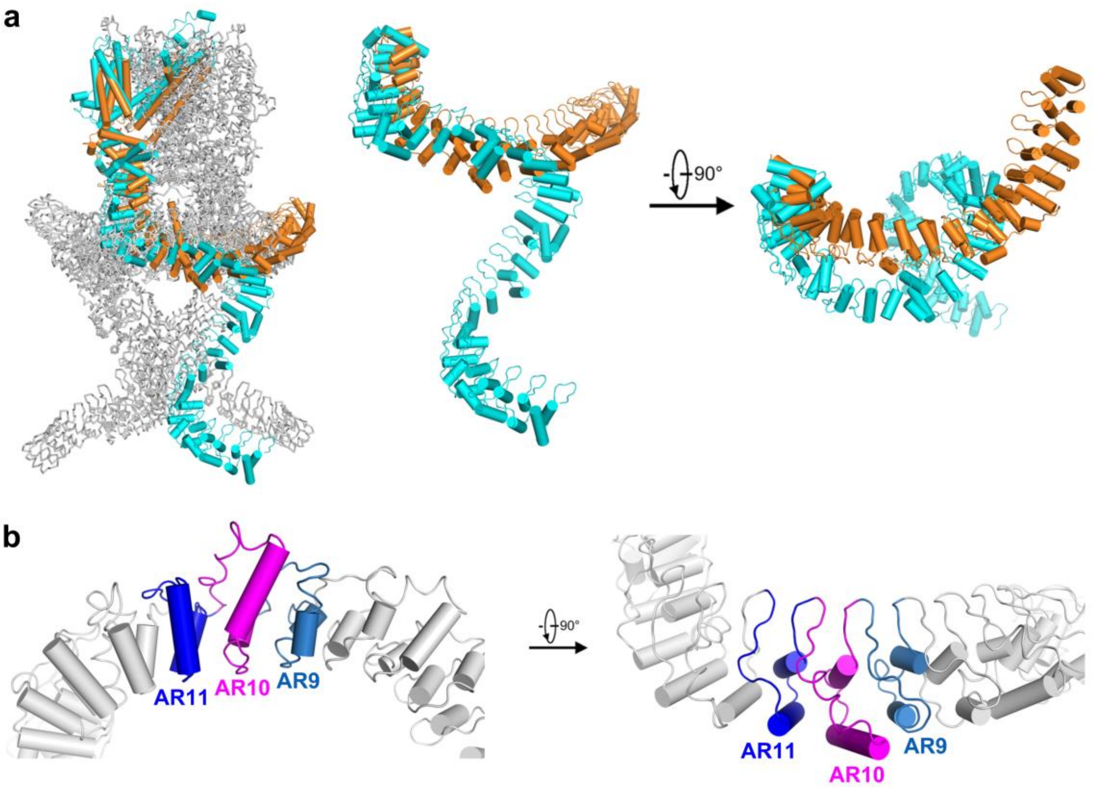

Supplementary Fig. S4 Structure comparison of the ARD between dTRPA1-A and dNOMPC.

a. Overall trend for the seventeen ARs observed in the dTRPA1 state-1 structure is quite different from that in dNOMPC (PDB ID: 5VKQ). One subunit of dTRPA1-A and dNOMPC is coloured orange and cyan, respectively. Two perpendicular reviews of the ARD alignment are shown on the right. **b.** Zoomed view of AR10 in the state-1 structure of dTRPA1-A isoform. AR10 is the turning point for the overall trend of the ARD in dTRPA1-A. The outer helix of AR10 is dramatically tilted compared to AR9 and AR11.

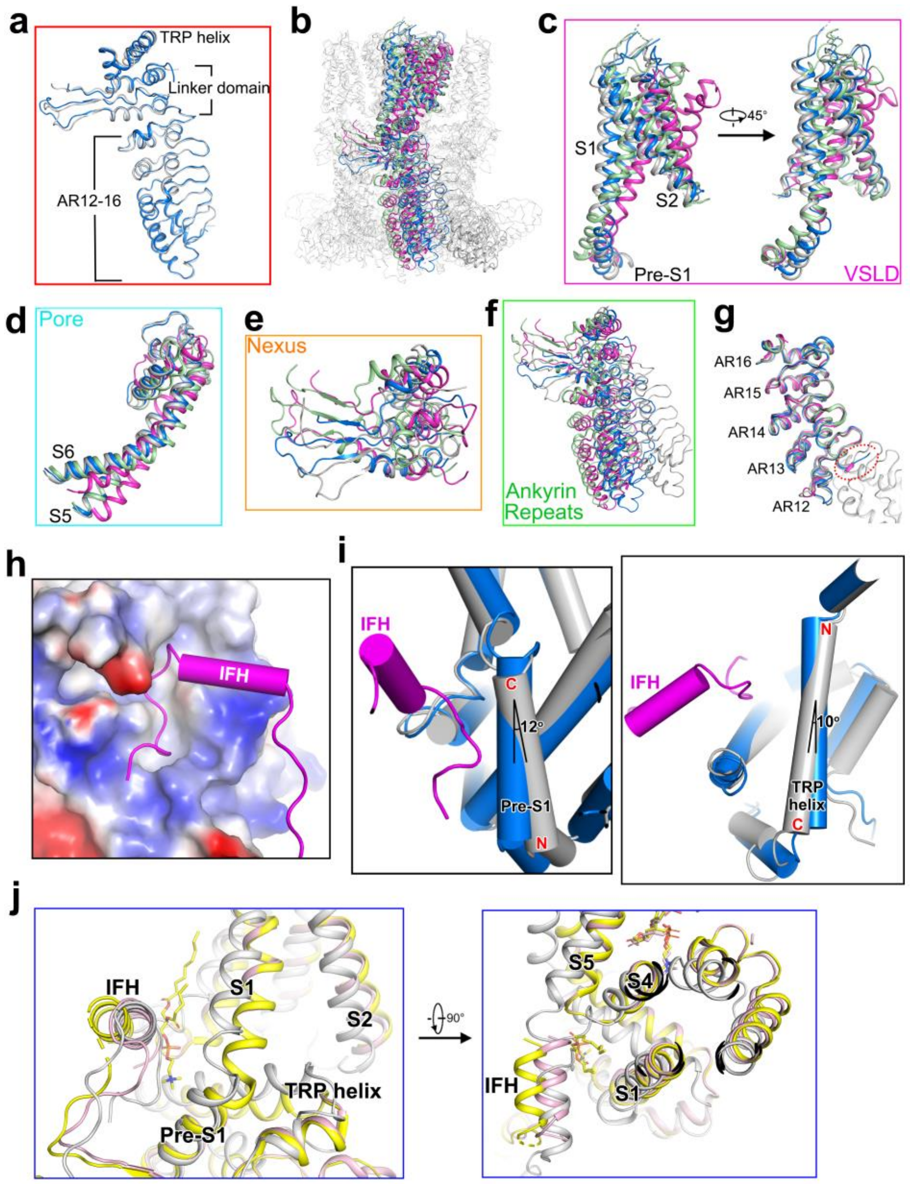

Supplementary Fig. S5 Structure comparison between dTRPA1-A and hTRPA1.

a. Structure alignments of the cytosolic domain alone between two states of dTRPA1-A. The nexus domain and the last five ARs aligns well between two states, suggesting that they may move in a rigid-body manner during state transitions. The state-1 and state-2 structures of dTRPA1-A are shown in grey and marine blue, respectively. **b.** Overview of the structure alignments between the two state structure of dTRPA1-A, the closed state of hTRPA1 (PDB ID: 6V9W, coloured green) and the activated state of hTRPA1 (PDB ID: 6V9X, coloured magenta). **c.** Structure alignments between dTRPA1-A and hTRPA1 for the VSLD. **d.** Structure alignments between dTRPA1-A and hTRPA1 for the pore region. **e.** Structure alignments between

dTRPA1-A and hTRPA1 for the nexus region. **f.** Structure alignments between dTRPA1-A and hTRPA1 for the ARD. **g.** Structure alignments of the ARD alone reveal that the AR12-AR16 have an almost identical architecture. The N-terminus of AR12 is highlighted in a red circle. **h.** IFH (coloured magenta) and the succeeding loop insert into a pocket formed by the surrounding helices. **i.** Rotations of the pre-S1 and TRP helices between two states. **j.** Structure alignments of the IFH in dTRPA1-A and hTRPA1. Compared with the agonist JT010-bound hTRPA1 structure (PDB ID: 6PQO, coloured yellow) and the BITC-bound hTRPA1 structure (PDB ID: 6PQP, coloured pink), the IFH in the state-1 structure of dTRPA1-A (coloured grey) packs more tightly to nearby helices. The lipid molecule observed in the interfacial cavity of the JT010-bound hTRPA1 structure is shown in sticks.

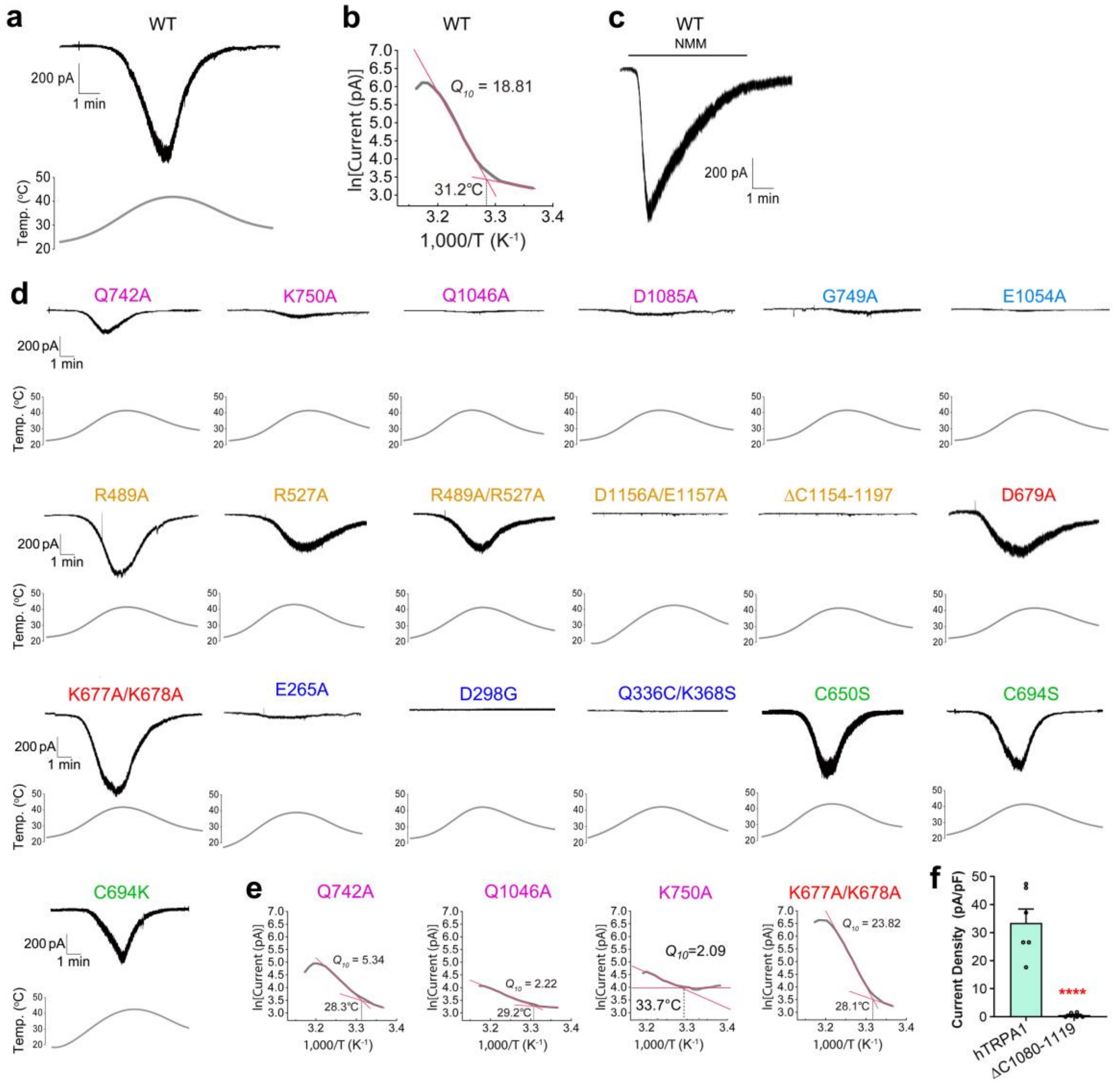

Supplementary Fig. S6 Electrophysiological characterizations of dTRPA1-A mutants.

a. Representative traces of whole-cell recordings for the WT dTRPA1-A under heat stimulus. Temperature (Temp.) is increased from 24 °C to 42 °C and then cooled down to 24 °C as indicated under the trace. **b.** Arrhenius plots of WT dTRPA1-A isoform. The Q_{10} value and transition temperature of each channel are indicated on the plot. **c.** NMM-induced whole-cell currents for the WT dTRPA1-A isoform. **d.** Representative traces of whole-cell recordings for the dTRPA1-A mutants under heat stimulus. Temperature (Temp.) is increased to 42 °C and then cooled to 24 °C as indicated under each trace. **e.** Arrhenius plots of the dTRPA1-A mutants. The Q_{10} value and transition temperature of each channel are indicated in the plot. **f.** hTRPA1 activation by AITC. Compared with the WT channel, the C-terminus truncation had

an abolished response to AITC treatment at a concentration of 200 μM. Independent experiments were repeated six times ($n = 6$) for each construct. Significances were determined using a two-tailed unpaired t-test. **** $P < 0.0001$. Data are presented as mean ± s.e.m.

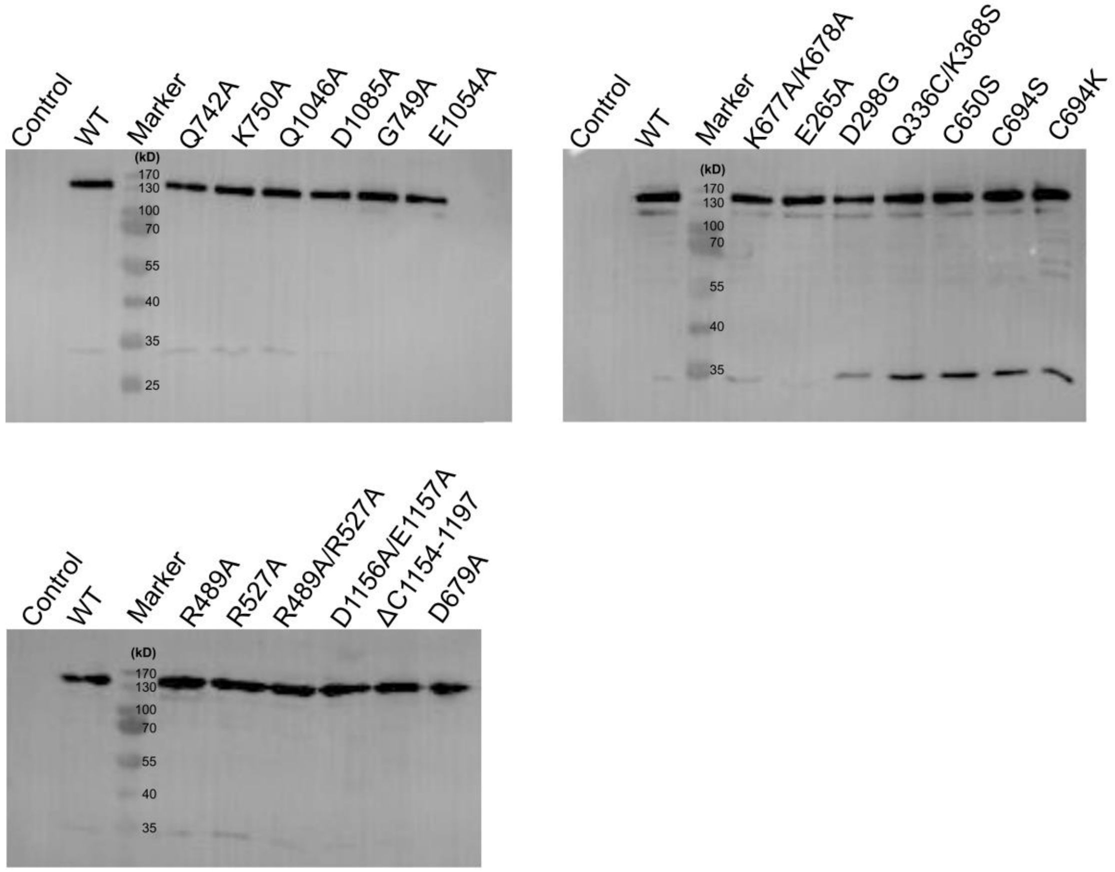

Supplementary Fig. S7 Expression of the WT dTRPA1-A isoform and the mutants in HEK293F cells.

Expression of WT dTRPA1-A isoform and all the mutants tested detected by western blot using anti-Flag tag antibody.

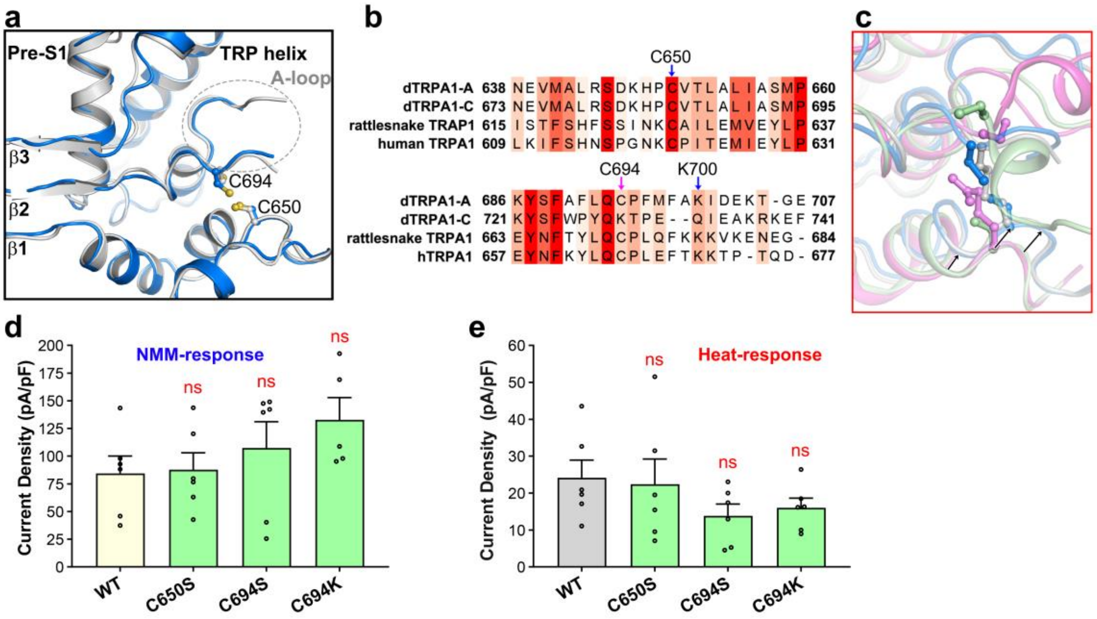

Supplementary Fig. S8 Reactive cysteine residues in dTRPA1-A.

a. Zoomed view of the structure alignment between two states of dTRPA1-A. State-1 is shown in grey and state-2 is shown in marine blue. Side chains of the key residues are shown in sticks. **b.** Sequence alignments between dTRPA1 isoforms and TRPA1 from human and rattlesnake. Shown here are the two segments containing the two reactive cysteine residues. **c.** Structure alignment of the nexus domain form hTRPA1 and dTRPA1-A alone. Side chains of the cysteine residues are shown in a ball-and-stick manner in the two state structure of dTRPA1-A, the closed state of hTRPA1 (PDB ID: 6V9W, coloured green) and the activated state of hTRPA1 (covalently modified by iodoacetamide, PDB ID: 6V9X, coloured magenta). Structural shifts for the first helix-turn-helix motif are indicated by black arrows. **d.** NMM-induced current densities at -60 mV for WT dTRPA1-A and the cysteine mutants. Independent experiments were repeated for at least four times for each construct (*n* = 6 for all, except C694K where *n* = 5). ns=not significant for WT versus mutants (one-way ANOVA with Dunnett's multiple comparisons test). Data are mean ± s.e.m. **e.** Heat-induced current densities for WT dTRPA1-A and the cysteine mutants. Independent experiments were repeated for at least four times for each construct (*n* = 6 for all). ns=not significant for WT versus mutants (one-way ANOVA with Dunnett's multiple comparisons test). Data are mean ± s.e.m.

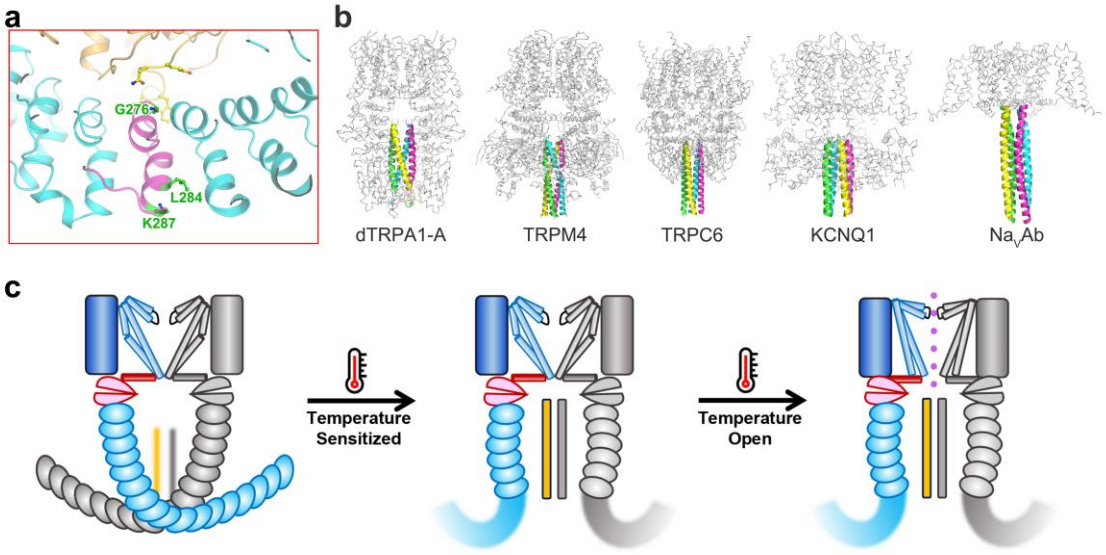

Supplementary Fig. S9 Model of the thermal activation of dTRPA1-A isoform. **a.** Locations of the three residues in AR6 of dTRPA1-A isoform that are essential to thermal activation. **b.** Representative four-helix bundle structures in dTRPA1-A (state-2), TRPM4 (PDB ID:5WP6), TRPC6 (PDB ID:5YX9), KCNQ1 (PDB ID:5VMS) and the BacNa$_V$ channel Na$_V$Ab (PDB ID:5HK7). **c.** A cartoon model for the thermal activation of dTRPA1-A isoform. For thermal activation, TRPA1 is first sensitized from state-1 to a conformation like the state-2 structure of dTRPA1-A, and opens through movements of the TRP and pre-S1 helices in the nexus region.

Supplementary Tables

Supplementary Table S1. Statistics of cryo-EM data collection, 3D reconstructions, model refinement and validation.

**Data Collection**		
EM equipment	FEI Titan Krios	
Voltage (kV)	300	
Detector	Gatan K2 Summit	
Pixel size (Å)	1.01	
Electron dose (e^-/Å^2)	50	
Defocus range (μm)	-1.5 ~ -2.3	
**3D Reconstruction**	**State-1**	**State-2**
Software	RELION 3.0	RELION 3.0
Number of Particles	74,515	184,926
Symmetry	C4	C4
Map Resolution (Å)	3.2	3.0
FSC Threshold	0.143	0.143
**Model Refinement**	**State-1**	**State-2**
Map Sharpening B-factor (Å^2)	-151.0	-161.3
Model Resolution (Å)	3.3	3.2
FSC Threshold	0.5	0.5
Protein residues	4,212	2,364
Side chains	3,812	2,340
CC mask	0.85	0.83
**Validation**	**State-1**	**State-2**
R.m.s. Deviations		
Bond lengths (Å)	0.009	0.008
Bond angles (°)	0.988	0.970
MolProbity Score	1.68	1.62
All-atom Clashscore	3.82	4.00
Rotamer Outliers (%)	0.38	0.51
Ramachandran plot		
Favored (%)	91.17	93.31
Allowed (%)	8.83	6.69
Outliers (%)	0	0

Supplementary Table S2. ***Q_{10}*** **values and transition temperatures for the WT dTRPA1-A channel and the mutants.**

Type of dTRPA1	Q_{10} value	Transition Temperature (°C)
WT	18.81	31.2
Q742A	5.34	28.3
K750A	2.09	33.7
Q1046A	2.22	29.2
D1085A	4.83	31.4
G749A	3.84	37.0
E1054A	4.62	34.0
R489A	8.34	30.4
R527A	23.69	34.3
R489A/R527A	4.96	34.9
D1156A/E1157A	2.11	32.4
ΔC1154-1197	2.10	39.4
D679A	12.51	37.0
K677A/K678A	23.82	28.1
E265A	1.39	31.0
D298G	3.08	29.5
Q336C/K368S	2.03	28.1
C650S	20.02	30.7
C694S	5.55	30.0
C694K	9.29	28.5

Supplementary Videos

Supplementary Video S1. A morph showing the structural transitions between state-1 and state-2 of dTRPA1-A isoform.
